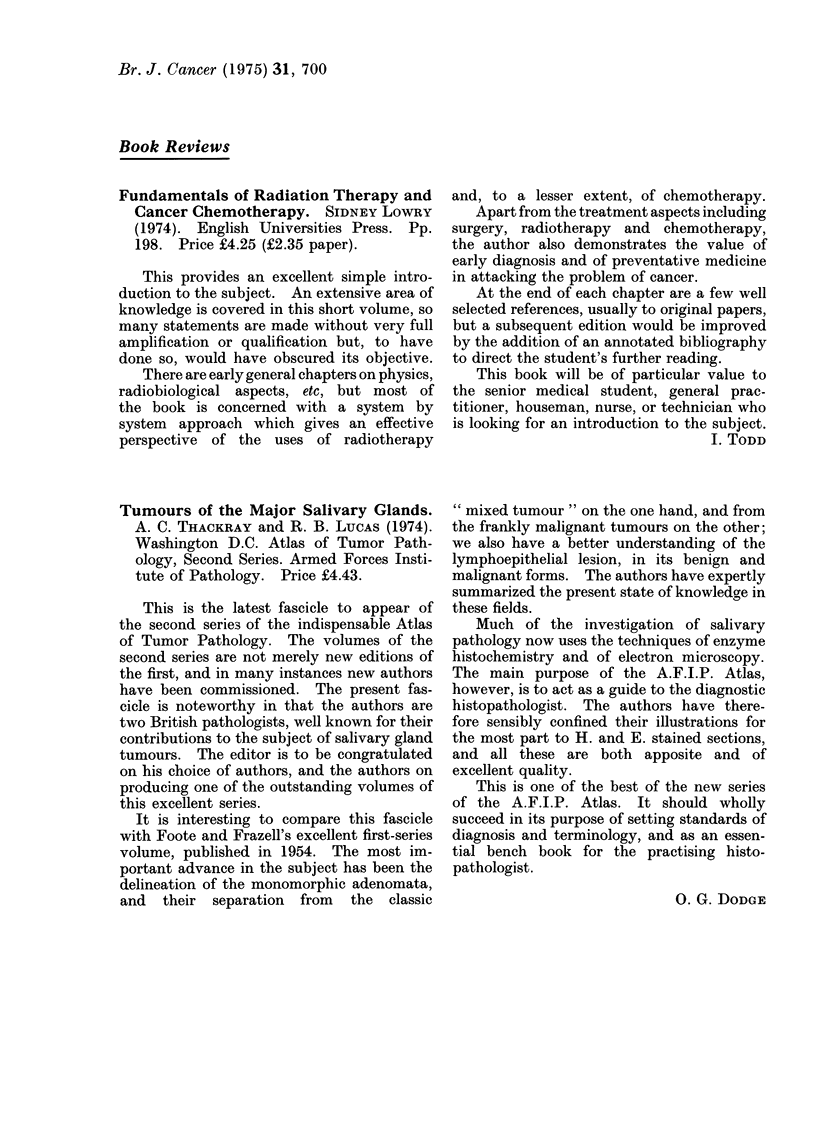# Tumours of the Major Salivary Glands

**Published:** 1975-06

**Authors:** O. G. Dodge


**Tumours of the Major Salivary Glands.** A. C. THACKRAY and R. B. LUCAS (1974). Washington D.C. Atlas of Tumor Pathology, Second Series. Armed Forces Institute of Pathology. Price £4.43.

This is the latest fascicle to appear of the second series of the indispensable Atlas of Tumor Pathology. The volumes of the second series are not merely new editions of the first, and in many instances new authors have been commissioned. The present fascicle is noteworthy in that the authors are two British pathologists, well known for their contributions to the subject of salivary gland tumours. The editor is to be congratulated on his choice of authors, and the authors on producing one of the outstanding volumes of this excellent series.

It is interesting to compare this fascicle with Foote and Frazell's excellent first-series volume, published in 1954. The most important advance in the subject has been the delineation of the monomorphic adenomata, and their separation from the classic "mixed tumour" on the one hand, and from the frankly malignant tumours on the other; we also have a better understanding of the lymphoepithelial lesion, in its benign and malignant forms. The authors have expertly summarized the present state of knowledge in these fields.

Much of the investigation of salivary pathology now uses the techniques of enzyme histochemistry and of electron microscopy. The main purpose of the A.F.I.P. Atlas, however, is to act as a guide to the diagnostic histopathologist. The authors have therefore sensibly confined their illustrations for the most part to H. and E. stained sections, and all these are both apposite and of excellent quality.

This is one of the best of the new series of the A.F.I.P. Atlas. It should wholly succeed in its purpose of setting standards of diagnosis and terminology, and as an essential bench book for the practising histopathologist.

O. G. DODGE